# Atherosclerosis and flow: roles of epigenetic modulation in vascular endothelium

**DOI:** 10.1186/s12929-019-0551-8

**Published:** 2019-08-07

**Authors:** Ding-Yu Lee, Jeng-Jiann Chiu

**Affiliations:** 1 Department of Biological Science and Technology, China University of Science and Technology, Taipei, 115 Taiwan; 2 Institute of Cellular and System Medicine, National Health Research Institutes, Miaoli, 350 Taiwan; 3 Institute of Biomedical Engineering, National Tsing Hua University, Hsinchu, 300 Taiwan; 4 Collage of Pharmacy, Taipei Medical University, Taipei, 110 Taiwan; 5 Institute of Biomedical Engineering, National Cheng Kung University, Tainan, 701 Taiwan; 6 Institute of Polymer Science and Engineering, National Taiwan University, Taipei, 106 Taiwan

**Keywords:** DNA methyltransferase, Endothelial cell, Epigenetic factor, Hemodynamic force, Histone deacetylase, Non-coding RNA

## Abstract

**Background:**

Endothelial cell (EC) dysfunctions, including turnover enrichment, gap junction disruption, inflammation, and oxidation, play vital roles in the initiation of vascular disorders and atherosclerosis. Hemodynamic forces, i.e., atherprotective pulsatile (PS) and pro-atherogenic oscillatory shear stress (OS), can activate mechanotransduction to modulate EC function and dysfunction. This review summarizes current studies aiming to elucidate the roles of epigenetic factors, i.e., histone deacetylases (HDACs), non-coding RNAs, and DNA methyltransferases (DNMTs), in mechanotransduction to modulate hemodynamics-regulated EC function and dysfunction.

**Main body of the abstract:**

OS enhances the expression and nuclear accumulation of class I and class II HDACs to induce EC dysfunction, i.e., proliferation, oxidation, and inflammation, whereas PS induces phosphorylation-dependent nuclear export of class II HDACs to inhibit EC dysfunction. PS induces overexpression of the class III HDAC Sirt1 to enhance nitric oxide (NO) production and prevent EC dysfunction. In addition, hemodynamic forces modulate the expression and acetylation of transcription factors, i.e., retinoic acid receptor α and krüppel-like factor-2, to transcriptionally regulate the expression of microRNAs (miRs). OS-modulated miRs, which stimulate proliferative, pro-inflammatory, and oxidative signaling, promote EC dysfunction, whereas PS-regulated miRs, which induce anti-proliferative, anti-inflammatory, and anti-oxidative signaling, inhibit EC dysfunction. PS also modulates the expression of long non-coding RNAs to influence EC function. i.e., turnover, aligmant, and migration. On the other hand, OS enhances the expression of DNMT-1 and -3a to induce EC dysfunction, i.e., proliferation, inflammation, and NO repression.

**Conclusion:**

Overall, epigenetic factors play vital roles in modulating hemodynamic-directed EC dysfunction and vascular disorders, i.e., atherosclerosis. Understanding the detailed mechanisms through which epigenetic factors regulate hemodynamics-directed EC dysfunction and vascular disorders can help us to elucidate the pathogenic mechanisms of atherosclerosis and develop potential therapeutic strategies for atherosclerosis treatment.

## Introduction

**Fig. 1:**
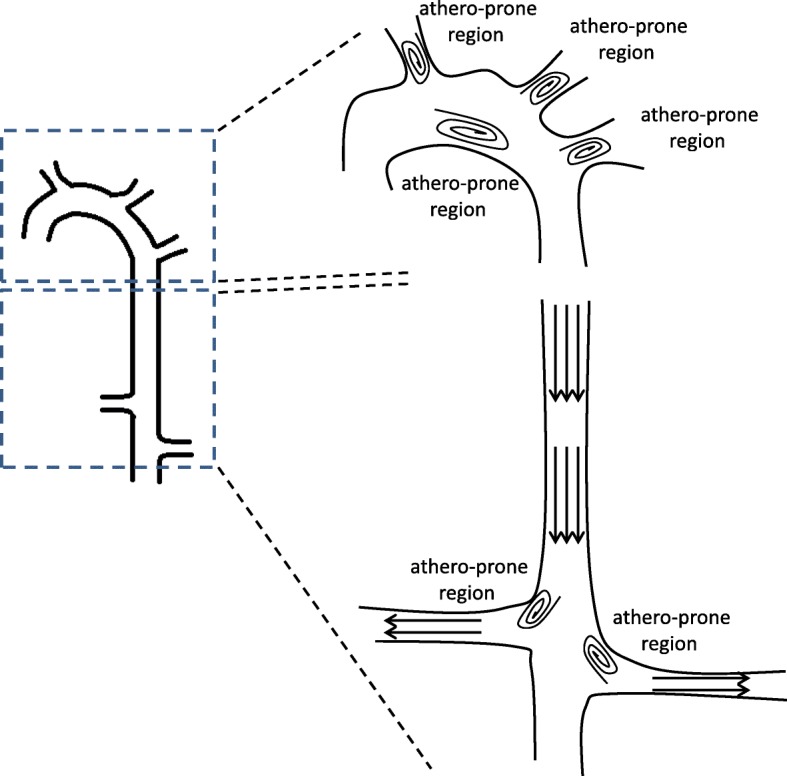
Distribution of hemodynamic forces, i.e., pro-atherogenic OS and atheroprotective PS, in the aortic trees. Pro-atherogenic OS and atheroprotective PS are two types of hemodynamic forces to affect EC function or dysfunction. Pro-atherogenic OS develops in the atherosclerosis-susceptible regions of aortic tree, e.g., the inner curvatures of aortic arch; carotid bifurcations; branch points of the coronary, infrarenal, and femoral arteries; and aorto-renal branches. In contrast, atheroprotective PS occurs in the atherosclerosis-protective regions of aortic tree, e.g., descending thoracic aorta and distal straight renal artery. The athero-prone regions are labeled in the figure.: pro-atherogenic OS;: atheroprotective PS

Vascular endothelial cells (ECs), which are located in the blood vessel wall and function to prevent vascular leakage and protect vascular vessels, are subjected to hemodynamic forces that can activate mechanotransduction and regulate homeostasis. Pro-atherogenic oscillatory shear stress (OS) and atheroprotective pulsatile shear stress (PS) are two vital hemodynamic forces that modulate EC dysfunction and function [1–3]. Pro-atherogenic OS serves as “bad flow” to activate various pro-atherogenic signaling pathways and gene expression, resulting in promotion of pathogenic conditions in ECs. In contrast, atheroprotective PS serves as “good flow” to induce many protective signaling pathways and gene expression, thereby maintaining normal physiological functions in ECs [1–3]. In the aortic circulation system, pro-atherogenic OS preferentially occurs in the specific regions of aortic trees, i.e., the inner curvatures of the aortic arch; carotid bifurcations; branch points of the coronary, infrarenal, and femoral arteries; and aorto-renal branches. These OS regions have been identified as atherosclerosis-susceptible regions in the aortic system (Fig. 1) [1–3]. Atheroprotective PS usually develops in the straight segments of aortic tree, i.e., the descending thoracic aorta and distal straight renal artery. These PS regions have been identified as athero-protective regions in the aortic system (Fig. 1) [1–3].

EC dysfunctions, including turnover enhancement, gap junction disruption, inflammation, and oxidation, have been found to play vital roles in the initiation of vascular disorders and atherosclerosis [4–7]. Turnover enhancement and gap junction disruption in ECs are pathogenic factors for atherosclerosis [1, 7, 8]. These responses can disrupt the intact structure of the endothelium to increase endothelial permeability and allow the penetration of lipoproteins and inflammatory monocytes to promote the progression of atherosclerosis [5–8]. Pro-atherogenic OS and atheroprotective PS exert differential effects on modulating cell proliferation and gap junctions in ECs. Pro-atherogenic OS induces EC proliferation and increases turnover rates by inducing DNA synthesis and cyclin and cyclin-dependent kinase expression and inhibiting p21_CIP1_ expression [9, 10]. In contrast, PS inhibits EC proliferation via induction of p21_CIP1_ to inhibit DNA synthesis, resulting in EC arrest at the G_0_/G_1_ phase [11]. In vivo, ECs in disturbed flow regions with pro-atherogenic OS have higher mitotic rates than the ECs in the straight segments of aorta with atheroprotective PS in the rabbit thoracic aorta [12]. Guo et al. [13] demonstrated that the differential effects of atheroprotective PS and pro-atherogenic OS on the cell cycle are mediated by AKT/mammalian target of rapamycin (mTOR)/p70 signaling pathways. Moreover, hemodynamic forces have been found to regulate junctional proteins, including connexins (Cx) and vascular endothelial (VE)-cadherin, to regulate endothelial permeability [14, 15]. Pro-atherogenic OS induces discontinuous distributions of VE-cadherin and β-catenin, whereas atheroprotective PS induces continuous distributions of these proteins [14]. Pro-atherogenic OS also induces discontinuous Cx43 at the EC periphery [15]. In vivo studies have further demonstrated that VE-cadherin is highly expressed at EC borders in the descending thoracic aorta (PS region), but is rarely expressed in the aortic arch (OS region) [14].

EC inflammation is an additional pathogenic factor for atherosclerosis [4–7, 16]. ECs have been found to increase the expression of pro-inflammatory chemotactic molecules, e.g., monocyte chemoattractant protein-1 (MCP-1), and adhesion molecules, e.g., intercellular adhesion molecule-1 (ICAM-1), vascular cell adhesion molecule-1 (VCAM-1), and E-selectin/P-selectin, to recruit monocytes for adhesion to and penetration into vessel walls, thereby initiating the progression of atherosclerosis [4–7, 16]. Pro-atherogenic OS and atheroprotective PS have opposite effects on modulating these pro-inflammatory genes to regulate EC dysfunction and function. Pro-atherogenic OS induces the sustained expression or activation of transcription factors, e.g., nuclear factor (NF)-κB, to induce the expression of pro-inflammatory genes and activate atherogenic signaling in ECs. In contrast, atheroprotective PS only transiently induces or even inhibits the expression of these pro-inflammatory genes and the activation of atherogenic signaling in ECs [1, 17–20].

EC oxidation is also a pathogenic factor for atherosclerosis [4–7]. ECs can modulate intracellular superoxide and antioxidant enzymes to regulate atherogenic responses in the progression of atherosclerosis [21]. Pro-atherogenic OS induces a sustained increase of intracellular superoxide to enhance oxidative stress or reactive oxygen species (ROS) levels to damage blood vessels, whereas atheroprotective PS induces antioxidant enzymes, i.e., superoxide dismutase (SOD), heme oxygenase-1, and NADPH quinine oxidoreductase 1 (NQO1), to protect blood vessels [22, 23]. In addition to the modulatory effects of atheroprotective PS and pro-atherogenic OS on EC proliferation, inflammation, and oxidation, atheroprotective PS also activates protective signaling to maintain EC physiological function. PS induces the expression or activation of various atheroprotective signaling molecules, including endothelial nitric oxide synthase (eNOS), prostaglandin I_2_, nitric oxide (NO), glutathione peroxidase (Gpx), and glutathione reductase [1, 24, 25]. Taken together, these studies suggest that hemodynamic forces, i.e., pro-atherogenic OS and atheroprotective PS, exert differential effects on modulating EC function and dysfunction, subsequently regulating disease and health.

**Fig. 2:**
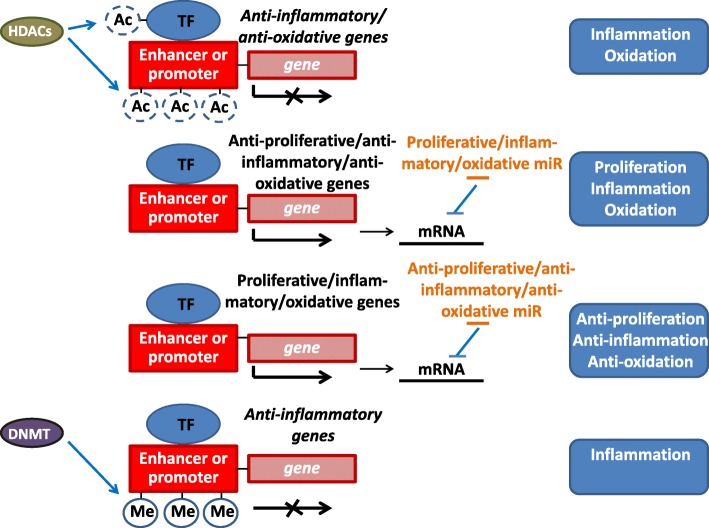
Epigenetic regulation of HDACs, non-coding RNAs, and DNMTs in vascular function and dysfunction. HDACs deacetylate not only the transcriptional factor, but also DNA regions of promoter or enhancer, to repress the expressions of anti-inflammatory or anti-oxidative genes. Proliferative, oxidative, and pro-inflammatory miRs target anti-proliferative, anti-oxidative, and anti-inflammatory mRNAs to drive proliferative, oxidative, and inflammatory signaling, respectively. In contrast, anti-proliferative, anti-oxidative, and anti-inflammatory miRs target the respective mRNAs to drive anti-proliferative, anti-oxidative, and anti-inflammatory signaling, respectively. DNMTs methylate DNA regions of promoter or enhancer to inhibit the expression of anti-inflammatory genes to elicit inflammatory signaling. Ac: acetylation; TF: transcription factor; Me: methylation

Epigenetic modulation is defined as any stable and heritable change in gene expression or cellular function without changes in DNA sequences [26]. These modifications, which include covalent and noncovalent modifications of DNA, as well as histone tails, affect changes in chromatin structure and gene expression. DNA deacetylation, DNA methylation, and RNA-based mechanisms are the three main forms of epigenetic modulation. DNA deacetylation and methylation are catalyzed by specific enzymes, i.e., histone deacetylases (HDACs) and DNA methyltransferases (DNMTs), to regulate gene expression. RNA-based mechanisms are directed by non-coding RNAs to regulate gene expression [27–31]. Recent studies have shown that these epigenetic factors, including HDACs [27], non-coding RNAs [28–30], and DNMTs [31], play vital roles in epigenetic regulations of vascular function and dysfunction (Fig. 2).

This review focus on discussing (1) the effects of hemodynamic forces, i.e., OS and PS, on modulating the expression and activation of epigenetic factors in ECs and (2) the roles of hemodynamics-modulated epigenetic factors in regulating mechanotransduction, including signaling molecules, transcription factors, and gene expression, involved in atherogenic and atheroprotective signaling in ECs. Moreover, the relationship between hemodynamics-modulated epigenetic factors and vascular disorders is also discussed. This article summarizes the evidence that epigenetic factors are vital factors for modulating hemodynamics-directed EC function and dysfunction, and provides insights into the functional roles of epigenetic factors in the development of atherosclerosis in responses to flow.

### HDACs in hemodynamics-directed EC function and dysfunction

#### Classification and function of HDACs

Histone deacetylation is a common modification affecting chromosome packaging and DNA transcription. HDACs are specific enzymes that function to deacetylate the ε-N-acetyl lysine amino acid on histones to modulate the chromatin structure involved in DNA replication or silencing [32]. Hyperacetylation is related to upregulation of transcription, whereas hypoacetylation is associated with downregulation of transcription [32]. In addition to histone deacetylation, HDACs also interact with nonhistone proteins, i.e., various signaling molecules and transcription factors, to repress their functions [27]. Because most functions of HDACs, i.e., histone deacetylation and transcription factor repression, are processed only in the nucleus, the localization of HDACs is vital for evaluating their functions [27]. HDACs in mammalian cells are classified into four groups depending on their sequence similarity: class I HDACs (HDAC-1, − 2, − 3, and − 8), class II HDACs (IIa: HDAC-4, − 5, − 7, and − 9, and IIb: HDAC-6, − 10), class III HDACs (sirtuins (Sirts): Sirt1–7), and class IV HDACs (HDAC-11). Classes I, II, and IV HDACs are zinc-dependent enzymes, whereas class III HDACs are NAD + -dependent enzymes [33–35].

Class I HDACs are nuclear enzymes. HDAC-1, − 2, and − 8 are generally found only in the nucleus, whereas HDAC-3 shuttles between the nucleus and cytoplasm [33–35]. Inhibition studies have demonstrated that class I HDACs play vital roles in modulating cell proliferation and survival [33–35]. HDAC-1 and -2 can be recruited by transcription factors, including Sp1, Sp3, p53, NF-κB, and YY1, to form the multiprotein corepressor Sin3, nucleosome-remodeling HDAC, and CoREST [33–35], which repress the transcriptional activity and cellular functions of these transcription factors. HDAC-3 is involved in two complexes, i.e., nuclear receptor corepressor (NCoR or NCOR1) and silencing mediator of retinoic acid and thyroid hormone receptor (SMRT or NCOR2) complexes. NCoR and SMRT further recruit class II HDACs as bridges for HDAC-3 to enhance NCoR/SMRT/HDAC-3 activity and repress specific transcription factors, i.e., myocyte enhancer factor 2 (MEF2), which modulates MEF2-directed cell functions [33–35]. In addition, the phosphorylation of HDAC-1 (at S393, S421, and S423), HDAC-2 (at S394, S422, and S424), and HDAC-3 (at S424) can enhance their activity and further repress transcription factor function [33–35].

Class II HDACs are located in both the nucleus and cytosol and can be phosphorylated to regulate their nuclear/cytosolic shuttling [36, 37]. The functions of class II HDACs are related to cell inflammation and migration. As described above, class IIa HDACs (HDAC-4, − 5, − 7, and − 9) can collaborate with NCoR/SMRT/HDAC-3 to repress MEF2 transcriptional activity and related cellular functions, including inflammation. In addition to MEF2, class IIa HDACs can also associate with other transcription factors to repress their transcriptional activity [33–35]. Moreover, class IIa HDACs can be phosphorylated to enhance their binding to 14–3-3 proteins, induce nuclear export, and rescue the repression of transcription factors and downstream genes. Thus, nuclear-cytoplasmic shuttling of class IIa HDACs can be regulated by various kinases and phosphatases involved in signaling transduction to modulate the functions of class IIa HDACs and various transcription factors [36, 37]. Class IIb HDACs (HDAC-6 and -10) also shuttle between the nucleus and cytoplasm, but exist primarily in the cytoplasm [33–35]. HDAC-6 functions as a α-tubulin or cortactin deacetylase to regulate microtubule- and actin-dependent cell motility. Moreover, HDAC-6 can form aggresomes to clear misfolded proteins [33–35, 38].

Class III HDACs (Sirt1–7) can transfer an acetyl group from lysine to the cofactor nicotinamide adenine dinucleotide (NAD+) to generate O-acetyl ADP-ribose and nicotinamide, which serve as feedback inhibitors of the enzymatic reaction. Sirt1, 6, and 7 locate in the nucleus, Sirt3, 4, and 5 locate in the micochondria, and Sirt2 locates in the cytosol [39]. Sirts have various functions in protein modification, including ADP-ribosyltransferase activity and removing fatty-acyl groups from lysine residues. They are involved in energy metabolism, inflammation, genome stability, and aging [39].

The function of the class IV HDAC, HDAC-11, remains unclear. A few studies have suggested that this HDAC has a role in evolutionary conservation and the balance between immune activation and tolerance [40].

#### Roles of HDACs in vascular function

HDACs have been found to play important roles in vascular biology [27]. The crucial vascular functions of HDACs have been elucidated in knockdown studies. Class I HDACs (HDAC-1, − 2, and − 3) are related to cardiac morphogenesis and endothelial survival. Montgomery et al. [41] demonstrated that HDAC-1 and -2 modulate cardiac morphogenesis, growth, and contractility. Cardiac-specific knockout of either HDAC1 or HDAC2 does not have significant effects on the cardiac phenotype. Cardiac-specific deletion of both HDAC-1 and HDAC-2 induces neonatal lethality and cardiac abnormalities, including cardiac arrhythmias and dilated cardiomyopathy. Zampetaki et al. [42] found that lentivirus-mediated HDAC-3 silencing in mice induces disruption of the basement membrane and rupture of blood vessels, resulting in a lethal phenotype. Class II HDACs (HDAC-5, − 7, and − 9) are also associated with cardiovascular function. Chang et al. [43] demonstrated that knockout of HDAC-5 or − 9 in mice results in cardiac hypertrophy. They also found that inhibition of HDAC-7 in mice induces the loss of EC-cell interactions and the rupture of blood vessels, resulting in embryonic lethality [44]. Class III HDACs have been shown to have protective functions in blood vessels, preventing atherosclerosis [45]. These studies have suggested that HDACs play vital roles in cardiovascular development and function.

#### Roles of HDACs in shear-modulated EC function versus dysfunction

**Fig. 3:**
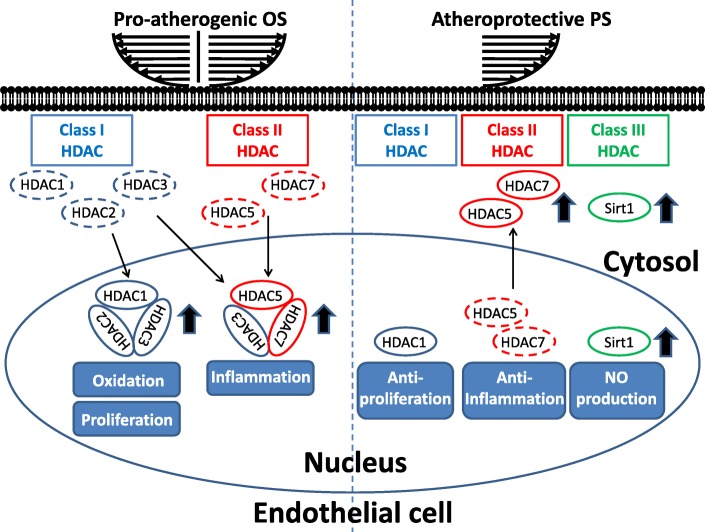
Roles of HDACs in modulating hemodynamics-regulated EC dysfunctions, including proliferation, inflammation, and oxidation. Pro-atherogenic OS induces expression and nuclear accumulation of both class I (HDAC-1, − 2, and -3) and class II HDACs (HDAC-5 and -7). Moreover, OS further enhances the formation of HDAC-1/HDAC-2/HDAC-3 and HDAC-3/HDAC-5/HDAC-7 heterocomplexes to promote proliferation, inflammation, and oxidation. In contrast, atheroprotective PS induces phosphorylation-dependent nuclear export of class II HDACs to decrease HDAC levels in the nucleus to inhibit their effects on proliferation, inflammation, and oxidation. On the other hand, PS induces the expression of Class III (Sirt1) to enhance NO production

Several studies have demonstrated that HDACs play important roles in modulating hemodynamics-induced EC function and dysfunction (Table 1). Studies in the laboratories of Shyy and Xu first demonstrated the roles of HDACs in flow-regulated EC function. Shyy and colleagues [46] found that PS induces HDAC-1 activation, resulting in the deacetylation of p53 and enhancement of p21 expression. Additionally, Xu and colleagues [47] demonstrated that HDAC3 is an important factor regulating PS-induced cell differentiation from stem cells into ECs through the Flk-1/phosphatidylinositol 3-kinase/Akt/HDAC3/p53/p21 pathway. In our previous studies, we identified the roles of class I and class II HDACs in modulating cellular functions, including proliferation, oxidation, and inflammation, in ECs in response to pro-atherogenic OS and atheroprotective PS (Fig. 3) [48]. We found that pro-atherogenic OS can induce the expression and nuclear accumulation of both class I (HDAC-1, − 2, and − 3) and class II HDACs (HDAC-5 and -7) in ECs. OS also can enhance the phosphorylation of class I HDACs to increase their activity. Krüppel like factor 2 (KLF-2) and NF-E2-related factor 2 (Nrf2), two important transcription factors that direct anti-inflammatory and antioxidant responses, govern approximately 70% shear-responsive genes [49]. OS-induced HDAC-1, − 2, and − 3 can associate with Nrf2 to repress its binding to antioxidant response element to inhibit the expression of antioxidant NQO1 [48]. In addition, OS-induced HDAC-3 can cooperate with HADC-5 and -7 (class II HADCs) to associate with MEF2 and abolish the expression of anti-inflammatory KLF-2. Moreover, OS-induced HDAC-1, − 2, and − 3 can modulate cell cycle regulators, i.e., upregulation of cyclin A and downregulation of p21_CIP1_, to promote EC proliferation. In contrast, atheroprotective PS has no effect on inducing the expression and nuclear accumulation of both class I (HDAC-1, − 2, and − 3) and class II (HDAC-5 and -7) HDACs, but induces phosphorylation-dependent nuclear export of class II HDACs. These PS-induced effects decrease HDAC levels in the nucleus to inhibit their repressive effects on transcription factors (Fig. 3) [48].

**Table 1:** Roles of HDACs in hemodynamics-modulated endothelial function and dysfunction

	HDACs	Class	Localization	Affected molecule	EC dysfunction/function	Ref
Pro-atherogenic OS	HDAC-1↑	I	Nucleus↑	Nrf2 (deacetylation)/NQO1↓	Oxidation	48
				CyclinA ↑ /p21↓	Proliferation	48
	HDAC-2↑	I	Nucleus↑	Nrf2 (deacetylation)/NQO1↓	Oxidation	48
				CyclinA ↑ /p21↓	Proliferation	48
	HDAC-3↑	I	Nucleus↑	Nrf2 (deacetylation)/NQO1↓	Oxidation	48
				CyclinA ↑ /p21↓	Proliferation	48
				AKT (phosphorylation)	Survival	42
	HDAC-4↑	II	Nucleus↑	MEF2 (deacetylation)/KLF2 ↑ VCAM ↓	Inflammation	48
	HDAC-5↑	II	Nucleus↑	MEF2 (deacetylation)/KLF2 ↓ VCAM↑	Inflammation	48
	HDAC-7↑	II	Nucleus↑	MEF2 (deacetylation)/KLF2 ↑ VCAM↓	Inflammation	48
Atheroprotective PS	HDAC-1	I	Nucleus/Cytosol	p53 (deacetylation)/p21↑	Antiproliferation	46
	HDAC-3	I	Nucleus/Cytosol	MEF2 (acetylation)/KLF2 ↑ VCAM↓	Anti-inflammation	48
			?	p53 (deacetylation)/p21↑	Differentiation into ECs	47
	HDAC-5	II	Cytosol↑	MEF2 (acetylation)/KLF2 ↑ VCAM↓	Anti-inflammation	48
				MEF2 (acetylation)/KLF2 ↑ eNOS ↑	NO production	50
	HDAC-6↑	II	Cytosol↑	α-tubulin	Migration	51
	HDAC-7	II	Cytosol↑	MEF2 (acetylation)/KLF2 ↑ VCAM↓	Anti-inflammation	48
	Sirt1↑	III	?	eNOS (deacetylation)	NO production	52

In vivo OS conditions created by the U-clip stenosed model and native circulation in rats were used to confirm that both class I and class II HDACs are highly expressed in ECs in response to OS. In addition, EC proliferation is found in the OS region of the rat U-clip stenosed model in vivo. Administration of the class I inhibitor VPA in experimental rats abolishes OS-induced EC proliferation in vivo. Our studies showed that hemodynamic forces, i.e., pro-atherogenic OS and atheroprotective PS, modulate the expressions or nuclear/cytosolic shuttling of class I (HDAC-1, − 2, and − 3) and class II (HDAC-5 and -7) HDACs to regulate anti-inflammatory and antioxidant signaling by altering the acetylation of transcription factors, including MEF2 and Nrf-2, in EC nuclei, which affect their transcription activities and the expression of downstream anti-inflammatory *KLF-2* and antioxidant *NQO1* genes. Moreover, pro-atherogenic OS-induced class I HDACs are involved in modulating EC proliferative signaling through regulation of cell cycle-related proteins, including cyclin A and p21 (Fig. 3) [48].

Other studies have also elucidated the roles of class I, class II, and class III HDACs in hemodynamics-modulated EC function and dysfunction (Table 1). Zampetaki et al. [42] found that orbital shaker-generated OS can induce serine/threonine phosphorylation of HDAC-3 to modulate endothelial survival and integrity via AKT activation. Additionally, Wang et al. [50] found that PS can induce phosphorylation-dependent nuclear export of HDAC-5 in ECs through a calcium/calmodulin-dependent pathway. PS-induced nuclear export of HDAC-5 decreases the ability of HDAC-5/MEF2 to enhance MEF2 acetylation and transcriptional activity and induce the expressions of KLF-2 and eNOS, which are key mediators involved in flow-mediated anti-inflammatory and -protective functions. Wang et al. [51] indicated that PS increases the expression of HDAC-6 to deacetylate tubulin and induce EC migration. Class III HDACs (e.g., Sirt1) have also been shown to have roles in modulating shear-dependent EC function. For example, Chen et al. [52] indicated that PS increases the expression and activity of Sirt1. PS-induced Sirt1 can associate with eNOS to induce eNOS deacetylation. These results suggested that PS-induced Sirt1 (a class III HDAC) increases NO bioavailability in ECs.

In addition to hemodynamics-modulated EC function and dysfunction, HDACs are also involved in the progression of vascular disorders, including atherosclerosis [53, 54]. HDACs are involved in various processes in atherosclerotic formation, including blood glucose and plasma lipid elevation, monocyte accumulation and migration, foam cell formation, vascular smooth muscle cell (SMC) phenotype switch, fibrous cap formation, plaque disruption, and thrombosis [54]. Kee et al. [55] showed that the HDAC inhibitor trichostatin A activates KLF-4 to inhibit balloon injury-induced neointimal hyperplasia. Other studies have also linked different classes of HDACs to vascular disorders, including atherosclerosis. For example, Findeisen et al. [56] showed that endovascular injury of the mouse femoral artery induces the expression of class I HDACs and the formation of neointima. Treatment with the HDAC inhibitor scriptaid inhibits injury-induced neointima formation. However, knockdown of HDAC3 induces EC apoptosis, as shown by increased extensive membrane blebs and cytosolic nucleosomes and enhanced annexin V staining, thereby accelerating neointima formation [42]. Usui et al. [57] found that the class IIa HDAC inhibitor MC1568 inhibits not only the activation of HDAC4 in the neointima region but also the formation of neointimal hyperplasia in a mouse carotid ligation model. Class III HDACs have been shown to prevent atherosclerosis in blood vessels [45, 58, 59]. Overexpression of Sirt1 in the endothelium in ApoE-deficient (ApoE^−/−^) mice induces the expression of eNOS, represses the expression of adhesion molecules, and subsequently inhibits the progression of atherosclerosis [58]. Haploinsufficiency of Sirt6 in ApoE^−/−^ mice has been found to promote atherogenesis [59]. These studies indicate that HDACs play vital roles in the progression of vascular disorders, including atherosclerosis.

### Non-coding RNAs in hemodynamics-directed EC function versus dysfunction

#### The biosynthesis and function of microRNAs (miRs)

MiRs, which are small noncoding RNAs (18–22 nucleotides in length), have emerged as new post-transcriptional repressors that function by binding to the mRNA of target genes to initiate their degradation and translational repression [28–30]. The transcriptional expression of miRs is regulated by transcription factors, including retinoic acid receptor (RAR) and KLF-2 [30, 60, 61]. These transcription factors show variations in acetylation or expression, affecting their binding activity for promoters or enhancers of miRs to modulate miR transcription in ECs. Subsequently, miRs are transcribed from DNA to generate primary miRs (pri-miRs) in the nucleus by RNA polymerase II or III. Pri-miRs are processed by a processor complex composed of DGCR8 and Drosha into ~ 60–100-nucleotide precursor miRs (pre-miRs) with a 3′ overhang hairpin structure. Pre-miRs are then transported into the cytoplasm by exportin-5. In cytosol, pre-miRs are processed by the RNA-induced silencing complex, which contains Ago2 and Dicer, to remove the hairpin structure to form a 22-nucleotide miR/miR* duplex. The miR strand of the miR/miR* duplex is processed into mature miR, whereas the miR* strand of miR/miR* is degraded. Mature miR further cooperates with Dicer and other associated proteins to form an miR-induced silencing complex and base pairs with 6–8 nucleotides within the 3′-untranslated region (UTR) of target genes, which exerts important functions in modulating target genes, including mRNA degradation or translation repression [28–30].

#### Roles of miRs in vascular function

The effects of miRs on the regulation of gene expression are involved in various physiological functions in health and disease [28–30]. miRs have been found to play important roles in various organs and tissues, including heart, muscle, liver, and kidney [62]. Blockage of miR biosynthesis in zebrafish and mice by suppression of important miR processers, including Dicer, has demonstrated that miRs modulate cardiovascular functions. Dicer-null zebrafish embryos show disrupted blood circulation and severe defects in cardiac development [63]. Dicer-deficient mice, generated by homologous recombination in embryonic stem cells, also show severe defects in blood vessel formation and maintenance [64]. Cardiac-specific deletion of Dicer modulates miR expression to induce dysregulation of adhesion proteins, cardiac remodeling, and heart failure [65, 66]. In addition, several miRs have been found to regulate cardiovascular function. For example, *miR-1* prevents high-fat diet-induced endothelial permeability in ApoE^−/−^ mice [67]. In contrast, knockout of *miR-133a* in mice results in dilated cardiomyopathy and heart failure [68]. Knockdown of *miR-126* in zebrafish induces the loss of vascular integrity and promotes hemorrhage during embryogenesis [69]. Similarly, deletion of *miR-126* in mice results in severe systemic edema, multifocal hemorrhage, and ruptured blood vessels throughout embryogenesis [70].

#### Transcriptional regulation of miRs by hemodynamic forces in ECs

Hemodynamic forces, i.e., OS and PS, exert differential effects on modulating miR expression and function in ECs [29, 30]. Chien and collaborators [71, 72] first used an miR microarray to examine the expression profiles of miRs in ECs in response to atheroprotective PS in vitro. Eight upregulated miRs and thirteen downregulated miRs have been found in ECs in response to PS. Among these shear-regulated miRs, *miR-19a* and *miR-23b* have been found to be robustly upregulated by PS to modulate EC growth arrest [71, 72]. In addition, Ni et al. [73] also used an miR microarray to compare miR profiles in ECs subjected to OS versus PS in vitro; they showed that *miR-663* was the miR with the highest expression in OS-treated ECs. OS-induced *miR-663* activates pro-inflammatory responses in ECs. Davies and colleagues [74] used an miR microarray to compare the expression profiles of miRs in the endothelium of atherosusceptible regions versus atheroprotected regions in normal adult swine in vivo. Among 1139 miRs, they found seven downregulated miRs and twenty seven upregulated miRs in the endothelium of atherosusceptible regions (OS regions) in comparison to atheroprotected regions (PS regions). Additionally, *miR-10a* was identified as the miR with the lowest expression in the endothelium of atherosusceptible regions (OS regions) versus atheroprotective regions (PS regions). They also demonstrated that knockout of *miR-10a* activates IκB/NF-κB-mediated pro-inflammatory responses in ECs in vitro. Moreover, Son et al. [75] used an miR microarray to identify the miR profiles in ECs subjected to OS versus PS in vivo using a partially ligated mouse model. They further used in vitro flow conditions to confirm the in vivo results and found that *miR-712* was the most robustly upregulated miR in ECs in response to OS in both in vivo and in vitro. These studies suggested that the expression of miRs is differentially modulated by different hemodynamic forces, including pro-atherogenic OS and atheroprotective PS, to affect EC function.

**Fig. 4:**
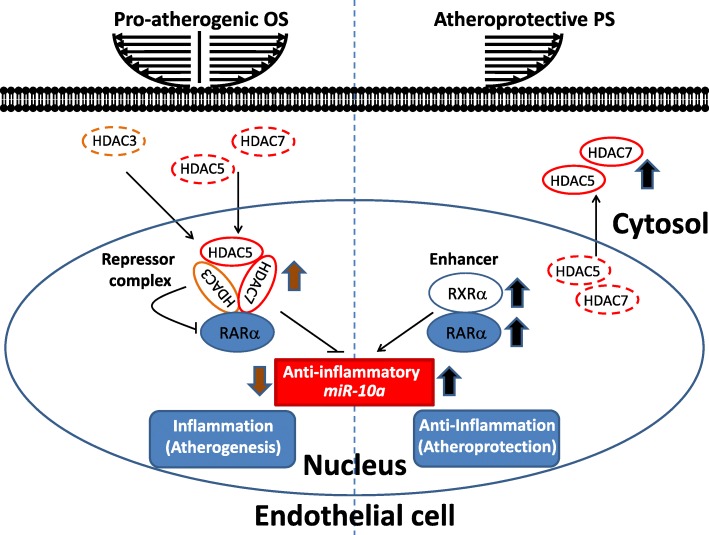
Schematic diagram of regulatory machinery for modulating atherosclerosis. Atheroprotective PS induces the expression, nuclear accumulation, and association of RARα (director) and RXRα (enhancer) to promote RARα/RARE binding and *miR-10a* expression in ECs. PS-induced *miR-10a* targets pro-inflammatory transcription factor GATA6 to repress its expression to inhibit downstream VCAM-1 expression. PS-induced RARα/*miR-10a* signaling elicits anti-inflammatory signaling in ECs. In contrast, pro-atherogenic OS induces the association of RARα with HDAC-3/− 5/− 7 (repressors) to repress RARα-directed *miR-10a* signaling to induce pro-inflammatory responses in ECs

Transcriptional initiation, which is regulated by transcription factors, is important for modulating miR expression in ECs in response to hemodynamic forces. Hemodynamic forces, i.e., OS and PS, modulate the activity and expression of transcription factors, i.e., RARα and KLF-2, to regulate miR expression [30, 60, 61]. Our previous study identified the mechanisms through which hemodynamic forces modulate transcriptional activity of RARα, resulting in changes in the expression of *miR-10a* and affecting anti-inflammatory signaling and cellular functions in ECs (Fig. 4) [60, 76]. Our results in an in vitro flow system showed that atheroprotective PS induces the expression, nuclear accumulation, and association of RARα and retinoid X receptor (RXR) α (an enhancer of RARα) to promote the binding of RARα to RA-responsive elements in the enhancer region of *miR-10a*, resulting in increased *miR-10a* expression in ECs. PS-induced *miR-10a* further targets the 3′-UTR of pro-inflammatory GATA6 to repress its expression, leading to inhibition of VCAM-1. In contrast, pro-atherogenic OS induces the association of RARα with HDAC-3/− 5/− 7 (repressors of RARα) to repress RARα-directed *miR-10a* signaling. These in vitro results were further confirmed in vivo by *en face* staining of aortic arch (OS region) versus the straight thoracic aorta (PS region) in rats. Likewise, the transcription factor KLF-2 is regulated by hemodynamic forces to modulate the expression of miRs. KLF-2 is a shear-sensitive transcription factor whose expression is upregulated by atheroprotective PS, but downregulated by pro-atherogenic OS in vivo and in vitro [77]. Nicoli et al. [61] demonstrated that KLF-2 is induced by PS to modulate *miR-126* and stimulate angiogenesis in zebrafish. The transcriptional start site of *miR-126* is predicted to contain a KLF-2 binding site in humans. Moreover, Chien and colleagues showed that KLF-2 also modulates PS-induced *miR-23b* [78]. In addition to *miR-126* and *miR-23b*, KLF-2 is also predicted to transcriptionally regulate several miRs, including *miR-10a*, *miR-30a*, *miR-143*, *miR-145*, and *miR-150* [30]. These studies have suggested that the expression or acetylation of transcription factors, e.g., RARα or KLF-2, can be differentially regulated by pro-atherogenic OS and atheroprotective PS to modulate the transcriptional expression of miRs.

#### Roles of miRs in shear-modulated EC function versus dysfunction

MiRs have also been shown to play essential roles in modulating hemodynamics-induced EC functions, including proliferation, inflammation, and oxidation. One group of miRs, including *miR-19a*, *miR-23b*, *miR-101*, *miR-155*, and *miR-126-5p*, is involved in hemodynamics-modulated EC proliferation (Table 2) [29, 30]. As described above, *miR-19a* and *miR-23b* are upregulated by PS to modulate EC growth arrest by degrading cell cycle regulatory proteins, including cyclin D1 and E2F1 [71, 72]. PS induces KLF-2-dependent biosynthesis and expression of *miR-23b*, leading to cyclin-dependent kinase-activating kinase complex repression and cell cycle suppression [78]. Chen et al. [79] found that *miR-101* is upregulated by PS to target proliferative signaling molecules, including mTOR, to repress its expression and induce cell arrest in ECs. Weber et al. [80] demonstrated that PS induces *miR-155* to inhibit EC proliferation and migration. In addition, Schober et al. [81] showed that pro-atherogenic OS inhibits *miR-126-5p* to induce the expression of its direct target Dlk1, resulting in enhanced EC proliferation and promotion of atherosclerosis.

**Table 2:** Roles of non-coding RNAs in hemodynamics-modulated endothelial function and dysfunction

	Non-coding RNA	Classification	Direct target	Affected molecule	EC dysfunction/function	Ref
Pro-atherogenic OS	*miR*					
	*miR-126-5p*↓	Antiproliferative miR	Dlk1↑		Proliferation	81
	*miR-10a*↓	Anti-inflamatory miR	GATA6↑	VCAM-1↑	Inflammation	60, 76
			TAK1, βTRC↑	NF-κB p65 ↑	Inflammation	74
				E-sel, VCAM-1, ↑	Inflammation	74
				IL-6, IL-8, MCP-1↑	Inflammation	74
	*miR-92a*↑	Inflammatory miR	KLF2↓	IL-6, MCP-1 ↑,TM↓	Inflammation	82
	*miR-663*↑	Inflammatory miR	?	KLF4↓	Inflammation	73
	*miR-712*↑	Inflammatory miR	↓TIMP	Souluble TNF-α↑	Inflammation	75
	*miR-21*↑	Inflammatory miR	↓PPARα	AP-1/VCAM-1, MCP-1↑	Inflammation	83
	*miR-34a*↑	Inflammatory miR	?	NF-κB/VCAM-1, ICAM-1↑	Inflammation	84
	*miR-21*↑	Oxidative miR	SOD↓	?	Oxidation	30
	miR17*↑	Oxidative miR?	SOD, GPx, Trx2↓	?	Oxidation?	30
	*miR-92a*↑	NO-related miR	KLF2↓	eNOS↓	NO repression	82
Atheroprotective PS	*miR*					
	*miR-19a*↑	Anti-proliferative miR	CyclinA↓	?	Antiproliferation	71
	*miR-23b*↑	Anti-proliferative miR	E2F↓	Rb phosphorylation↓	Antiproliferation	72
			?	CAK complex (repression)	Antiproliferation	78
	*miR-101*↑	Anti-proliferative miR	mTOR↓	?	Antiproliferation	79
	*miR-155*↑	Anti-proliferative miR	MYLK↓	RhoA	Antiproliferation Anti-proliferation	80
	*miR-10a*↑	Anti-inflamatory miR	GATA6↓	VCAM-1↓	Anti-inflammation	60, 76
	*miR-146a*↑	Anti-inflammatory miR	IRAK↓	NF-κB signaling↓	Anti-inflammation	85
	*miR-708*↑	Anti-inflammatory miR	IKK-γ↓	NF-κB signaling↓	Anti-inflammation	85
	*miR-451*↑	Anti-inflammatory miR	IL-6R↓	NF-κB signaling↓	Anti-inflammation	85
	*miR-98*↑	Anti-inflammatory miR	CHUK↓	NF-κB signaling↓	Anti-inflammation	85
	*miR-92a*↓	Anti-inflammatory miR	KLF2↑	TM ↑	Anti-inflammation	82
		NO-related miR	KLF2↑	eNOS↑	NO production	82
	*miR-21*↑	NO-related miR	PTEN↓	eNOS (phosphorylation)↑	NO production	87
	*lncRNAs*					
	*STEEL*↓	Turnover-related lnRNA		eNOS, KLF-2↑	Turnover, migration	93
					NO production???	
	*MANTIS*↑	Aligment-related lnRNA		Smad6, Sox18	Cell alignment	94
					Angiogenesis	
	*LASSIE*↑	Aligment-related lnRNA			Cell alignment	95
					Cell-cell interaction	
	*LISPR1*↑	Migration-related lnRNA		S1PR1 signaling↑	Migration	96

Another group of miRs, including *miR-10a*, *miR-92a*, *miR-633*, *miR-712*, *miR-21*, *miR-34a*, *miR-146a*, *miR-708*, *miR-451*, and *miR-98*, are involved in shear-modulated EC inflammation (Table 2) [29, 30]. As described above, knockdown of *miR-10a* in ECs activates NF-κB signaling [74]. Moreover, *miR-10a* can be differentially regulated by hemodynamic forces, including PS and OS, to modulate pro-inflammatory GATA6/VCAM-1 via the association of RARα with RXRα and HDACs [60, 76]. Wu et al. [82] demonstrated that *miR-92a* regulates hemodynamics-dependent EC inflammation and function. Moreover, PS downregulates, whereas OS upregulates, *miR-92a* expression to modulate KLF-2 expression in ECs. Shear-modulated *miR-92a* can further regulate the expression of eNOS and thrombomodulin (TM) to affect EC function. Ni et al. [73] showed that *miR-663* can be induced by pro-atherogenic OS to activate pro-inflammatory responses in ECs. Son et al. [75] found that *miR-712* is upregulated by OS to inhibit tissue inhibitor of metalloproteinase 3, enhance the release of soluble tumor necrosis factor-α and the adhesion of monocytes, and activate pro-inflammatory responses in ECs. They further demonstrated that treatment with a *miR-712* antagonist inhibits the progression of atherosclerosis. Zhou et al. [83] found that *miR-21* can be induced by OS to bind to the 3′-UTR of peroxisome proliferator-activated receptor (PPAR) α for degradation. OS-induced PPARα degradation decreases its inhibitory effect on the transcription factor activator protein-1 and enhances the expression of downstream VCAM-1 and MCP-1 to activate pro-inflammatory responses in ECs. Fan et al. [84] found that *miR-34a* is upregulated by OS, but downregulated by PS. Moreover, OS-induced *miR-34a* enhances the expression of ICAM-1 and VCAM-1 through NF-κB signaling, consequently promoting monocyte adhesion to ECs. Our recent study demonstrated that PS promotes the expression of anti-inflammatory miRs, including *miR-146a*, *miR-708*, *miR-451*, and *miR-98*. PS-induced *miR-146a*, *miR-708*, *miR-451*, and *miR-98* directly target interleukin-1 receptor-associated kinase, inhibitor of NF-κB kinase subunit-γ, interleukin-6 receptor, and conserved helix-loop-helix ubiquitous kinase genes, respectively, to inhibit NF-κB signaling [85]. Another group of miRs, including *miR-21*, miR-17*, and *miR-30b*, have been shown to regulate shear-dependent oxidative responses in ECs by mediating pro-oxidant or antioxidant enzymes, including SOD, GPx, thioredoxin-dependent peroxidase, and catalase (Table 2) [29, 30]. OS has been shown to induce the expression of *miR-21*, which targets the antioxidant SOD [30]. In addition, PS has been shown to inhibit the expression of *miR-17*, whose product miR-17* is proposed to target antioxidant SOD, GPx, and Trx2 [30]. Thus, *miR-21* and miR-17* may be involved in shear-modulated expression of antioxidant enzymes. The balance between NO and ROS is vital for maintaining EC function [86]. *MiR-21* and *miR-92a* have been found to be regulated by hemodynamic forces to modulate NO production. Weber et al. [87] demonstrated that *miR-21* is induced by PS, resulting in the phosphorylation of eNOS and the enhancement of NO production. Wu et al. [82] demonstrated that inhibition of *miR-92* by PS enhances eNOS expression and promotes NO production, whereas induction of *miR-92* by OS inhibits eNOS-directed NO production.

In addition to hemodynamics-modulated EC dysfunction, several miRs have been shown to be related to atherosclerosis. In our recent studies in ApoE^−/−^ mice, decreases in endothelial and serum *miR-10a* were found to be related to atherogenesis. Treatment of ApoE^−/−^ mice with pre-*miR-10a* or RARα/RXRα agonists can rescue *miR-10a* expression to inhibit the formation of atherosclerosis [76]. Schober et al. [81] showed that *miR-126-5p* suppresses the expression Dlk1 to prevent the progression of atherosclerosis. Loyer et al. [88] demonstrated that in vivo knockdown of *miR-92a* in LDLR^−/−^ mice inhibits endothelial inflammation and atherosclerosis progression. Moreover, Son et al. [75] demonstrated that treatment of ApoE^−/−^ mice with a *miR-712* antagonist inhibits the progression of atherosclerosis. Our recent results showed that treatment of mice with lentivirus carrying mature *miR-146a* blocks neointima formation in a mouse carotid artery ligation model [85].

#### Other non-coding RNAs relevant to shear-regulated EC dysfunction and atherosclerosis

In addition to miRs, recent studies indicate that other non-coding RNA categories, i.e., long noncoding RNAs (lncRNAs) and circular RNAs (circRNAs), also play important roles in the epigenetic regulation of endothelial function and atherosclerotic vascular diseases [89–92]. LncRNAs are non-coding RNAs with more than 200 nucleotides. They modulate various cellular processes in the nucleus and cytoplasm. In the nucleus, lncRNAs serve as histone modulators, enhancers or repressors, chromatin remodeling modulators, or transcription factor regulators to modulate transcription. In the cytoplasm, lncRNAs modulate mRNA stability, translation, and protein scaffold [89, 90]. Moreover, they can regulate protein phosphorylation and signaling transduction [89, 90]. On the other hand, circRNAs are circular non-coding RNAs, which are different from linear miRNA and lncRNAs. CircRNAs typically link the 5′ end to the 3′ end of RNAs to generate circular structure. They are also located in nucleus and cytoplasm to regulate cellular function. The function of circRNAs has been identified to regulate gene expression through transcriptional, post-transcriptional, and translational modulations [91, 92].

*Spliced-transcript endothelial-enriched lncRNA (STEEL)* and *MALAT1* were first identified to be shear-sensitive lncRNAs by Man et al. and Leisegang et al. (Table 2) [93, 94]. *STEEL* is an EC-specific lncRNA that enhances cell turnover and migration and has ability to promote blood vessel formation and maturation. Moreover, STEEL can transcriptionally regulate the expression of eNOS and KLF2, which are two major mediators of shear-responsive responses. *STEEL* performs epigenetic modulation in the transcriptional changes, including increased chromatin accessibility and histone methylation at the *eNOS* and *KLF2* promoters. The RNA and lncRNA of *STEEL* are decreased in ECs in response to atheroprotective PS, eliciting the upregulation of both KLF2 and eNOS. Overexpression or knockdown of KLF2 in ECs demonstrated that KLF2 has feedback inhibitiory effects on *STEEL* under atheroprotective PS condition [93]. *MANTIS* is an additional important lncRNA that facilitates endothelial angiogenic function. The level of *MANTIS* is enhanced by atheroprotective flow. Knockdown assay demonstrated that *MANTIS* can modulate shear-induced EC alignment and angiogenic sprounding [94]. On the other hand, Stanicek et al. [95] used RNA sequencing to identify that *LASSIE* is an atheroprotective flow-induced lncRNA. The shear-induced *LASSIE* is modulated by KLF2. Knockdown of *LASSIE* in ECs showed that *LASSIE* plays vital roles in cell-cell interactions and atheroprotective PS-induced EC alignment (Table 2). In addition, Josipovic et al. [96] found that *long intergenic noncoding RNA antisense to S1PR1 (LISPR1)* is also an atheroprotective flow-induced lncRNA. Its function has been found to regulate S1PR1 expression and S1P signaling pathway (Table 2). Moreover, several lncRNAs, including *H19* [97–99], *SENCR* [100], *MEG3* [101], and *RNCR3* [102], are related to atherosclerosis. *H19* expression has been found to be higher in human atherosclerotic lesion [103]. Moreover, high level of *H19* is also found in ApoE^−/−^ mice [98]. Overexpression of *H19* in ECs can induce upregulation of p38 MAPK and NF-κB and cellular proliferation [97]. In addition, the level of *H19* is higher in the plasma from human CAD patients with heart failure, as compared with that with normal cardiac function [99]. *SENCR* is a vascular lncRNA, which is enriched in ECs and SMCs. The expression of *SENCR* in ECs is identified to be downregulated in human CAD patients compared to healthy subjects [100]. Loss- and gain-of-function studies in ECs demonstrated that *SENCR* can modulate proliferation, migration, and tube formation of ECs. Moreover, *SENCR* has also been found to regulate the expression of pro-angiogenic genes, i.e., *CCL5*, *CEACAM1* and *CX3CL1*. *MEG3* is a lncRNA that is downregulated in human CAD tissues and proliferative ECs. *MEG3* has been identified to suppress EC proliferation through inhibiting the expression of cyclin D1, ki-67 and PCNA. In addition, *MEG3* also can inhibit the expressions of type I collagen, type V collagen and proteoglycan [101]. *RNCR3* is a lncRNA which is expressed in ECs and SMCs to regulate their proliferation, migration, and apoptosis. The expression of *RNCR3* has been found to be highly expressed in the atherosclerotic aortas of ApoE^−/−^ mice and human specimens. Knockdown of *RNCR3* in ApoE^−/−^ mice promotes the formation and enhances the levels of total cholesterol, triglycerides, and pro-inflammatory factors in blood [102].

Study on CircRNAs is a new field in vascular biology. Until now, only a few literatures correlate circRNAs to EC function and atherosclerosis. Dang et al. [104] used circRNA microarray to identify the expression profiles of hypoxia-stimulated ECs. They found 14 downregulated and 22 upregulated circRNAs in hypoxia-stimulated ECs. Among these circRNAs, *circ_0010729* was found to be significantly upregulated. Knockdown experiment of *circ_0010729* demonstrated that *circ_0010729* promotes EC proliferation and migration and inhibits EC apoptosis. On the other hand, Liu et al. [105] identified that cZNF609 was upregulated in high glucose- and hypoxia stress-treated ECs in vivo and in vitro. Knockdown and overexpression studies of *cZNF609* demonstrated that *cZNF609* can induce retinal vessel loss and pathological angiogenesis in vivo. Moreover, knockdown of *cZNF609* in ECs demonstrated that *cZNF609* can inhibit EC migration, tube formation, and protective effect against oxidative stress and hypoxia stress in vitro [105]. Holdt et al. [106] found that *circular antisense non-coding RNA in the INK4 locus (circANRIL)* impairs ribosome biogenesis and induces p53 activation to enhance apoptosis and reduce proliferation of SMCs and macrophage, and hence plays atheroprotective roles in vascular tissue. Overexpression of *circANRIL* in SMCs or macrophage can induce cell apoptosis and decrease cell proliferation. They also compared SMCs from different human CAD patients to demonstrate that high *circANRIL* expression induces apoptosis and reduces proliferation of SMCs. Song et al. [107] further elucidated the role of *circANRIL* in ECs in atherosclerotic rat model. Overexpression of *circANRIL* in atherosclerotic rat promotes apoptosis and inflammation of ECs and development of atherosclerotic plaques. Additionally, the levels of serum IL-1, IL-6, MMP-9 were increased in the *circANRIL*-expressed rats. They also suggested that *circANRIL* inhibition has a potential to be developed as therapeutic strategy for atherosclerosis treatment [107]. CircRNA in blood serum has also been proposed to be a diagnostic biomarker for CAD. Zhao et al. [108] used RNA microarray to compare peripheral blood circRNAs from 12 CAD patients and those from 12 healthy controls. They found 12 upregulated and 10 downregulated circRNAs in CAD patients. Among these circRNAs, they further identified *hsa_circ_0124644* as a potential biomarker for CAD. Moreover, Pan et al. [109] used microarray analysis to identify 18 upregulated and 6 downregulated circRNAs in blood serum of CAD patients in comparison to healthy subjects.

### DNMTs in hemodynamics-directed EC function versus dysfunction

#### Classification and function of DNMTs

In DNA methylation, a methyl group is added to the fifth carbon of a cytosine to form 5-methylcytosine (5mC) [110]. The regulation of most promoters in human genes is related to CpG islands, which are located in or near the promoter region and maintained in an unmethylated state to promote gene transcription [110]. In contrast, these DNA regions can be methylated to recruit methyl-CpG binding proteins and activate repressive machinery or inhibit the binding of transcription factors to promoters, resulting in chromatin compaction and inhibiting gene transcription [111]. DNA methylation plays essential roles in embryonic development and biological functions. Dysregulation of DNA methylation, i.e., hyper- or hypomethylation, results in various diseases, including cardiovascular diseases [112, 113].

DNA methylation is mainly regulated by DNMTs, which catalyze the addition of a methyl group to cytosine. De novo methyltransferases preferentially bind to unmethylated DNA to induce DNA methylation, whereas maintenance methyltransferases binds to hemimethylated DNA to induce DNA methylation. There are several DNMT isoforms, including DNMT1, DNMT3a, and DNMT3b, to be found [31, 114, 115]. DNMT1 is the most abundant DNMT in adult cells and acts primarily as a maintenance methylase. It has also been shown to have de novo methyltransferase activity. Deletion of DNMT1 induces genome hypomethylation and results in embryonic lethality [116]. DNMT3a and − 3b are classified as de novo methyltransferases. Deletion of both of DNMT3a and -3b results in early embryonic lethality. DNMT3a has been found to play roles in late development, whereas DNMT3b is involved in early development [117].

#### Roles of DNA methylation and DNMTs in vascular function

Aberrant DNA methylation and methyltransferase expressions are related to vascular disorders [118]. The reduction in genomic 5mC is observed in advanced atherosclerotic lesions in humans and ApoE^−/−^ mice. Moreover, hypomethylation of CpG islands is also found in the arteries of patients with atherosclerosis in comparison to that in control arteries [119]. However, the atheroprotective gene encoding estrogen receptor β is hypermethylated in atherosclerotic lesions of coronary arteries compared with that in normal control arteries [120]. In addition, the expressions of eNOS and vascular endotheilial growth factor receptor 2 (VEGF-R2) are repressed by methyl-CpG–binding domain protein 2 (MBD2), an MBD protein that binds to methylated DNA to mediate DNA methylation-dependent transcriptional repression, through directly binding to the methylated CpG elements in the promoters of these genes. Knockdown of MBD2 activates pro-angiogenic and protective signals, e.g., upregulation of VEGF-R2 and eNOS, to enhance EC angiogenesis and protect ECs against H_2_O_2_-induced apoptosis in vitro. Moreover, deletion of MBD2 in mice protects mice against hind-limb ischemia injury in vivo [121]. Thus, global DNA hypomethylation may be observed in atherosclerotic arteries, whereas specific DNA hypermethylation may occur in atheroprotective genes.

Notably, DNMTs are activated under pro-atherogenic conditions, i.e., a high-fat diet or high low-density lipoprotein (LDL) cholesterol levels. DNMT1 has been found to be overexpressed and activated in ApoE^−/−^ mice fed a high-fat diet [122]. Treatment of ECs with LDL cholesterol induces DNMT1 expression. In contrast, high levels of serum homocysteine, which acts as a source of methyl groups for methylation responses and has been shown to be a risk factor for EC inflammation and atherosclerosis, are found in patients with atherosclerosis [123]. Therefore, DNA methylation and DNMT play important roles in regulating vascular dysfunction.

#### Effects of hemodynamic forces on modulating DNMTs in ECs

Recent studies have shown that DNMTs, including DNMT1 and DNMT3a, are modulated by hemodynamic forces, i.e., OS and PS, to regulate inflammatory signaling (Table 3). Davies and coworkers [124] first showed that DNMT3a can be modulated by pro-atherogenic OS to regulate EC function. OS upregulates DNMT3a, which can bind to the promoter of *KLF-4*, a transcription factor that activates anti-inflammatory and antiproliferative responses in ECs, and induce DNA methylation of CpG islands in the *KLF-4* promoter, resulting in repression of *KLF-4* transcription. Shear-inhibition of KLF-4 further modulates downstream molecules, including NOS3, TM, and MCP-1. These in vitro results were confirmed in vivo by observation of the hypermethylation of the *KLF-4* promoter and downregulation of KLF-4 and NOS3 in the endothelium of OS regions in swine. At the same time, Jo et al. [125] and Chien et al. [126] demonstrated that DNMT1 is modulated by OS, resulting in induction of EC dysfunction. Moreover, DNMT-1 is upregulated by OS to regulate EC inflammation in vitro. A partial carotid ligation mouse model was used to generate OS in vivo, confirming that DNMT-1 is overexpressed in ECs in response to OS.

**Table 3:** Roles of DNMTs in hemodynamics-modulated endothelial function and dysfunction

	DNMTs	Target	Affected molecule	EC dysfunction/function	Ref
Pro-atherogenic OS	DNMT3a↑	KLF4↓	MCP-1↑	Inflammation	124
			Thrombomodulin↓	Inflammation	124
			NOS3↓	NO repression	124
	DNMT1↑	HoxA5↓	Monocyte adhesion-related genes↑	Inflammation	125
		?	VCAM-1↑	Inflammation	127
		?	ICAM-1↑	Inflammation	127
		CTGF↑	Monocyte adhesion-related genes↑	Inflammation	127
		?	PCNA↑	Proliferation	127
		CyclinA↑	?	Proliferation	127

Using both reduced representation bisulfite sequencing and microarray analysis, researchers found that hypermethylation occurred in the promoters of 11 mechanosensitive genes in ECs in response to OS. Among these 11 mechanosensitive genes, *HOXA5* is an important transcription factor that modulates inflammation. Thus, OS may mediate the methylation of promoters of mechanosensitive genes, including the transcription factor *HOX5*, to regulate OS-mediated pro-inflammatory responses [125]. Chien and colleagues [126] showed that OS induces not only the expression and nuclear accumulation of DNMT-1, but also the hypermethylation of DNA. Inhibition of DNMT-1 by 5-aza-2′-deoxycytidine (5Aza, also known as decitabine) suppresses OS-induced DNA hypermethylation. In concert with these findings, in vivo results showed that DNMT1 expression and DNA methylation are increased in OS regions of partially ligated rat carotid arteries [126]. Mechanistically, Zhang et al. [127] showed that OS-dependent induction of DNMT1 is modulated by integrin/Shc/focal adhesion kinase/extracellular signal-regulated kinase/mTOR/p70S6K signaling pathways. Moreover, OS-induced DNMT1 results in upregulation of cyclinA and connective tissue growth factor, which modulate EC proliferation and inflammation, respectively. These studies suggest that DNMT1 is involved in OS-induced EC dysfunction in vitro, including aberrant EC proliferation, inflammation, and NO repression.

DNMTs are also related to vascular disorders, including atherosclerosis. Jo and colleagues [125] showed that DNMT1 is correlated with atherosclerosis. In a partial carotid ligation mouse model, treatment of ApoE^−/−^ mice with 5Aza inhibits the formation of atherosclerosis. Zhang et al. [127] further used an ApoE^−/−^ mouse model to demonstrate that DNMT1 overexpression and DNA hypermethylation occur in the endothelium of atherosclerotic lesions. Silencing of DNMT-1 by adenovirus-mediated DNMT shRNA inhibits the expressions of EC dysfunction-related proteins, including proliferating cell nuclear antigen, VCAM-1, and ICAM-1, and blocks the development of atherosclerosis.

## Summary and conclusion

EC dysfunction, e.g., turnover enrichment, inflammation, and oxidation, is an important step for initiation of vascular disorder such as atherosclerosis. Vascular ECs are subjected to blood flow to activate mechanotransduction, which regulates EC function and dysfunction. Pro-atherogenic OS can modulate various signaling pathways to induce EC dysfunction and promote atherosclerosis. In contrast, atheroprotective PS can modulate various signaling pathways to inhibit EC dysfunction and protect against atherosclerosis.

Epigenetics has been emerged as a new field in vascular biology. In recent studies, epigenetic factors, including HDACs, non-coding RNAs, and DNMTs, have been shown to be involved in hemodynamic force-modulated EC function and dysfunction. In this review, we summarized current studies on the roles of these epigenetic factors in hemodynamics-modulated EC function and dysfunction, and hence atherosclerosis. Moreover, we discussed the detailed mechanisms by which mechanotransduction regulates epigenetic factors to affect EC function and dysfunction in response to various hemodynamic forces, i.e., pro-atherogenic OS and atheroprotective PS. Furthermore, we elucidated the relationship between epigenetic factors and vascular disorders, i.e., atherosclerosis. We discussed the mechanisms by which class I and II HDACs alter the expression of proliferative, pro-inflammatory, and oxidative signaling molecules to regulate EC function and dysfunction in response to differential hemodynamic forces. Pro-atherogenic OS induces the expression and nuclear accumulation of class I and II HDACs to induce EC dysfunction, whereas atheroprotective PS induces phosphorylation-dependent nuclear export of class II HDACs to inhibit EC dysfunction. In addition, class III HDACs, e.g., Sirt1, are induced by atheroprotective PS, resulting in acceleration of NO production.

We also discussed the novel mechanisms by which hemodynamic forces transcriptionally regulate miRs. Atheroprotective PS induces the expression, nuclear accumulation, and association of the hormone receptors RARα and RXRα to activate *miR-10a*-directed anti-inflammatory signaling. In contrast, pro-athergenic OS induces the association of HDAC-3/− 5/− 7 and RARα to form a repression complex and inhibit *miR-10a*-directed anti-inflammatory signaling. In addition, flow-modulated KLF-2 regulates several miRs, including *miR-126* and *miR-23b*. We summarized current studies showing how pro-atherogenic OS modulates miRs to activate proliferative, pro-inflammatory, and oxidative signaling and induce EC dysfunction, whereas atheroprotective PS modulates an array of miRs to drive antiproliferative, anti-inflammatory, anti-oxidative, and NO-related signaling and prevent EC dysfunction. Moreover, we provided new information that PS can modulate lncRNAs to regulate EC function, including cell turnover, migration, angiogenesis, and cell-cell interaction. Finally, pro-atherogenic OS has been shown to induce the expression of DNMT1 and DNMT3a and subsequently modulates EC dysfunction, i.e., proliferation, inflammation, and NO repression. All of these studies indicate that epigenetic factors, i.e., HDACs, miRs, lncRNAs, and DNMTs, are involved in hemodynamics-directed EC function and dysfunction and hence atherosclerosis. Understanding the relationship between epigenetic factors and EC function and dysfunction under pro-atherogenic or atheroprotective flow conditions will help to elucidate the pathogenic mechanisms of vascular disorders, such as atherosclerosis. In addition, the information provided in this review will help us to identify potential targets, which will facilitate the development of new strategies for the treatment of atherosclerosis.

## Data Availability

Not applicable.

## Abbreviations

5Aza: 5-aza-2′-deoxycytidine
5mC: 5-methylcytosine
ApoE^−/−^: Apolipoprotein E-deficient
CircRNAs: Circular RNAs
Cx: Connexins
DNMT: DNA methyltransferase
EC: Endothelial cell
eNOS: Endothelial nitric oxide synthase
Gpx: Glutathione peroxidase
HDAC: Histone deacetylase
ICAM-1: Intercellular adhesion molecule-1
KLF-2: Krüppel like factor 2
LDL: Low-density lipoprotein
lncRNAs: Long noncoding RNAs
MBD2: methyl-CpG–binding domain protein 2
MCP-1: Monocyte chemoattractant protein-1
MEF2: Myocyte enhancer factor 2
MiR: microRNA
mTOR: Mammalian target of rapamycin
NAD+: Nicotinamide adenine dinucleotide
NF: Nuclear factor
NO: Nitric oxide
NQO1: NADPH quinine oxidoreductase 1
Nrf2: NF-E2–related factor 2
OS: Oscillatory shear stress
PPAR: Peroxisome proliferator-activated receptor
Pre-miR: Precursor miR
Pri-miR: Primary miR
PS: Pulsatile shear stress
RAR: Retinoic acid receptor
ROS: Reactive oxygen species
RXR: Retinoid X receptor
Sirt: Sirtuin
SOD: Superoxide dismutase
TM: Thrombomodulin
UTR: Untranslated region
VCAM-1: Vascular cell adhesion molecule − 1
VE: Vascular endothelial
VEGF-R2: Vascular endotheilial growth factor receptor 2
